# Subtype-specific responses of hKv7.4 and hKv7.5 channels to polyunsaturated fatty acids reveal an unconventional modulatory site and mechanism

**DOI:** 10.7554/eLife.77672

**Published:** 2022-06-01

**Authors:** Damon JA Frampton, Koushik Choudhury, Johan Nikesjö, Lucie Delemotte, Sara I Liin

**Affiliations:** 1 Department of Biomedical and Clinical Sciences, Linköping University Linköping Sweden; 2 Science for Life Laboratory, Department of Applied Physics, KTH Royal Institute of Technology Solna Sweden; Universidad Nacional Autónoma de México Mexico; The University of Texas at Austin United States

**Keywords:** docosahexaenoic acid, electrophysiology, KCNQ, lipid, molecular dynamics simulations, omega 3, *Xenopus*

## Abstract

The K_V_7.4 and K_V_7.5 subtypes of voltage-gated potassium channels play a role in important physiological processes such as sound amplification in the cochlea and adjusting vascular smooth muscle tone. Therefore, the mechanisms that regulate K_V_7.4 and K_V_7.5 channel function are of interest. Here, we study the effect of polyunsaturated fatty acids (PUFAs) on human K_V_7.4 and K_V_7.5 channels expressed in *Xenopus* oocytes. We report that PUFAs facilitate activation of hK_V_7.5 by shifting the V_50_ of the conductance *versus* voltage (G(V)) curve toward more negative voltages. This response depends on the head group charge, as an uncharged PUFA analogue has no effect and a positively charged PUFA analogue induces positive V_50_ shifts. In contrast, PUFAs inhibit activation of hK_V_7.4 by shifting V_50_ toward more positive voltages. No effect on V_50_ of hK_V_7.4 is observed by an uncharged or a positively charged PUFA analogue. Thus, the hK_V_7.5 channel’s response to PUFAs is analogous to the one previously observed in hK_V_7.1–7.3 channels, whereas the hK_V_7.4 channel response is opposite, revealing subtype-specific responses to PUFAs. We identify a unique inner PUFA interaction site in the voltage-sensing domain of hK_V_7.4 underlying the PUFA response, revealing an unconventional mechanism of modulation of hK_V_7.4 by PUFAs.

## eLife digest

In order to carry out their roles in the body, cells need to send and receive electrical signals. They can do this by allowing ions to move in and out through dedicated pore-like structures studded through their membrane. These channels are specific to one type of ions, and their activity – whether they open or close – is carefully controlled. In humans, defective ion channels are associated with conditions such as irregular heartbeats, epileptic seizures or hearing loss.

Research has identified molecules known as polyunsaturated fatty acids as being able to control the activity of certain members of the K_V_7 family of potassium ion channels. The K_V_7.1 and K_V_7.2/7.3 channels are respectively present in the heart and the brain; K_V_7.4 is important for hearing, while K_V_7.5 plays a key role in regulating muscle tone in blood vessels. Polyunsaturated fatty acids can activate K_V_7.1 and K_V_7.2/7.3 but their impact on K_V_7.4 and K_V_7.5 remains unclear.

Frampton et al. explored this question by studying human K_V_7.4 and K_V_7.5 channels expressed in frog egg cells. This showed that fatty acids activated K_V_7.5 (as for K_V_7.1 and K_V_7.2/7.3), but that they reduced the activity of K_V_7.4.

Closely examining the structure of K_V_7.4 revealed that the fatty acids were binding to a different region compared to the other K_V_7 channels. When this site was made inaccessible, fatty acids increased the activity of K_V_7.4, just as for the rest of the family.

These results may help to understand the role of polyunsaturated fatty acids in the body. In addition, knowing how these molecules interact with channels in the same family will be useful for optimising a drug’s structure to avoid side effects. However, further research will be needed to understand the broader impact in a more complex biological organism.

## Introduction

K_V_7 voltage-gated potassium channels are expressed in several tissues, where they serve to attenuate excitability by conducting an outward K^+^ current. The five members of the family, K_V_7.1-K_V_7.5 (encoded by *KCNQ1-KCNQ5* genes), are important for human physiology, which is often emphasized in diseased states caused by dysfunctional channels. For instance, K_V_7.1 is predominantly expressed in cardiomyocytes, and mutations to this channel are a known risk factor for developing cardiac arrhythmia (Barhanin et al., 1996; Brewer et al., 2020; Sanguinetti et al., 1996; Tester and Ackerman, 2014). K_V_7.2 and K_V_7.3 are broadly expressed in neurons, where they form heterotetrameric K_V_7.2/7.3 channels, and mutations to these channels may give rise to epilepsy or chronic pain (Biervert et al., 1998; Nappi et al., 2020; Wang et al., 1998). K_V_7.4 is of particular importance in the auditory system where it forms homotetrameric channels responsible for a K^+^ conductance at the resting membrane potential of cochlear outer hair cells (OHCs) (Kharkovets et al., 2000; Kubisch et al., 1999; Rim et al., 2021). Mutations that perturb the trafficking or function of K_V_7.4 in OHCs are associated with a subtype of progressive hearing loss known as DFNA2 (Gao et al., 2013; Kharkovets et al., 2006; Kubisch et al., 1999; Rim et al., 2021). K_V_7.5 is more widely spread through the central nervous system and is particularly important in regulating excitability in the hippocampus (Fidzinski et al., 2015; Schroeder et al., 2000; Tzingounis et al., 2010). K_V_7.1, K_V_7.4, and K_V_7.5 are also expressed in various smooth muscle cells, such as vascular smooth muscle cells (VSMC), suggesting that several K_V_7 subtypes, including heterotetrameric K_V_7.4/7.5 channels, may contribute to the hyperpolarizing K^+^ current in VSMCs that promotes vasodilation by preventing Ca^2+^-dependent contraction (Mani et al., 2013; Ng et al., 2011; Stott et al., 2014; Bercea et al., 2021). The modulation of K_V_7.1 and K_V_7.2/7.3 channels by endogenous and pharmacological compounds has been extensively studied (Miceli et al., 2018; Wu and Larsson, 2020). However, less is known about the modulation of K_V_7.4 and K_V_7.5. Here, we studied the effects of a class of channel modulators, polyunsaturated fatty acids (PUFAs), on K_V_7.4 and K_V_7.5.

There is emerging evidence that suggests that PUFAs influence the physiology of tissues that express K_V_7.4 and K_V_7.5 channels. For instance, a number of studies have found an inverse relationship between hearing loss and the PUFA plasma concentration, suggesting that the risk of impaired hearing decreases with an increased dietary intake of ω–3 PUFAs such as docosahexaenoic acid (DHA) and eicosapentaenoic acid (EPA) (Curhan et al., 2014; Dullemeijer et al., 2010; Gopinath et al., 2010). Meta-analyses of randomized control trials have found that a multitude of beneficial cardiovascular outcomes, including antiinflammatory and hypotensive effects, are associated with an increased intake of PUFAs (AbuMweis et al., 2018; Miller et al., 2014). There are likely several mechanisms that contribute to these PUFA effects. For instance, the protective PUFA effect on hearing loss has been attributed to cerebrovascular effects, reasoning that PUFAs improve circulation to the cochlea (Curhan et al., 2014; Dullemeijer et al., 2010; Gopinath et al., 2010). PUFA-induced vasodilation has in part been attributed to the activation of Ca^2+^-dependent and ATP sensitive K^+^ channels by PUFAs (Hoshi et al., 2013; Limbu et al., 2018; Bercea et al., 2021). However, little is known about the putative direct contribution of K_V_7.4 and K_V_7.5 channels to PUFA effects. We find this open question interesting because PUFAs have been shown to facilitate activation of both K_V_7.1 and K_V_7.2/7.3 channels (Bohannon et al., 2019; Larsson et al., 2020; Liin et al., 2015; Liin et al., 2016; Taylor and Sanders, 2017). This PUFA-induced facilitation of activation is mediated through a lipoelectric mechanism in which the PUFA tail inserts into the outer leaflet of the lipid bilayer adjacent to the channel, whereupon the negatively charged carboxyl head group of the PUFA interacts electrostatically with positively charged arginines in the upper half of the voltage-sensing domain (VSD) of the channel (Liin et al., 2018; Yazdi et al., 2021). This electrostatic interaction facilitates the outward movement of the S4 helix, causing a shifted voltage dependence of channel opening toward more negative voltages. However, the effect of PUFAs on K_V_7.4 and K_V_7.5 remains unstudied. In this study, we therefore aimed to characterize the response of human K_V_7.4 and K_V_7.5 channels (henceforth referred to as hK_V_7.5 or hK_V_7.4) to PUFAs, in order to expand our understanding of how the K_V_7 family of channels responds to these lipids.

We report that PUFAs facilitate activation of hK_V_7.5 by shifting the voltage dependence of channel opening toward more negative voltages. Surprisingly, we find that PUFAs inhibit activation of the hK_V_7.4 channel by shifting the voltage dependence of channel opening toward more positive voltages. Thus, the hK_V_7.5 channel’s response to PUFAs is largely in line with the responses that have previously been observed in hK_V_7.1 and hK_V_7.2/7.3, whereas the hK_V_7.4 channel response is not. Providing a mechanistic explanation for this observation, we identify an unconventional inner PUFA site in the VSD of hK_V_7.4 underlying PUFA-induced inhibition of the activation of hK_V_7.4. Our study expands our understanding of how members of the hK_V_7 family respond to PUFAs and reveal subtype specific responses and sites to these lipids.

## Results

### The PUFA docosahexaenoic acid facilitates activation of hK_V_7.5, but inhibits activation of hK_V_7.4

**Figure 1.**
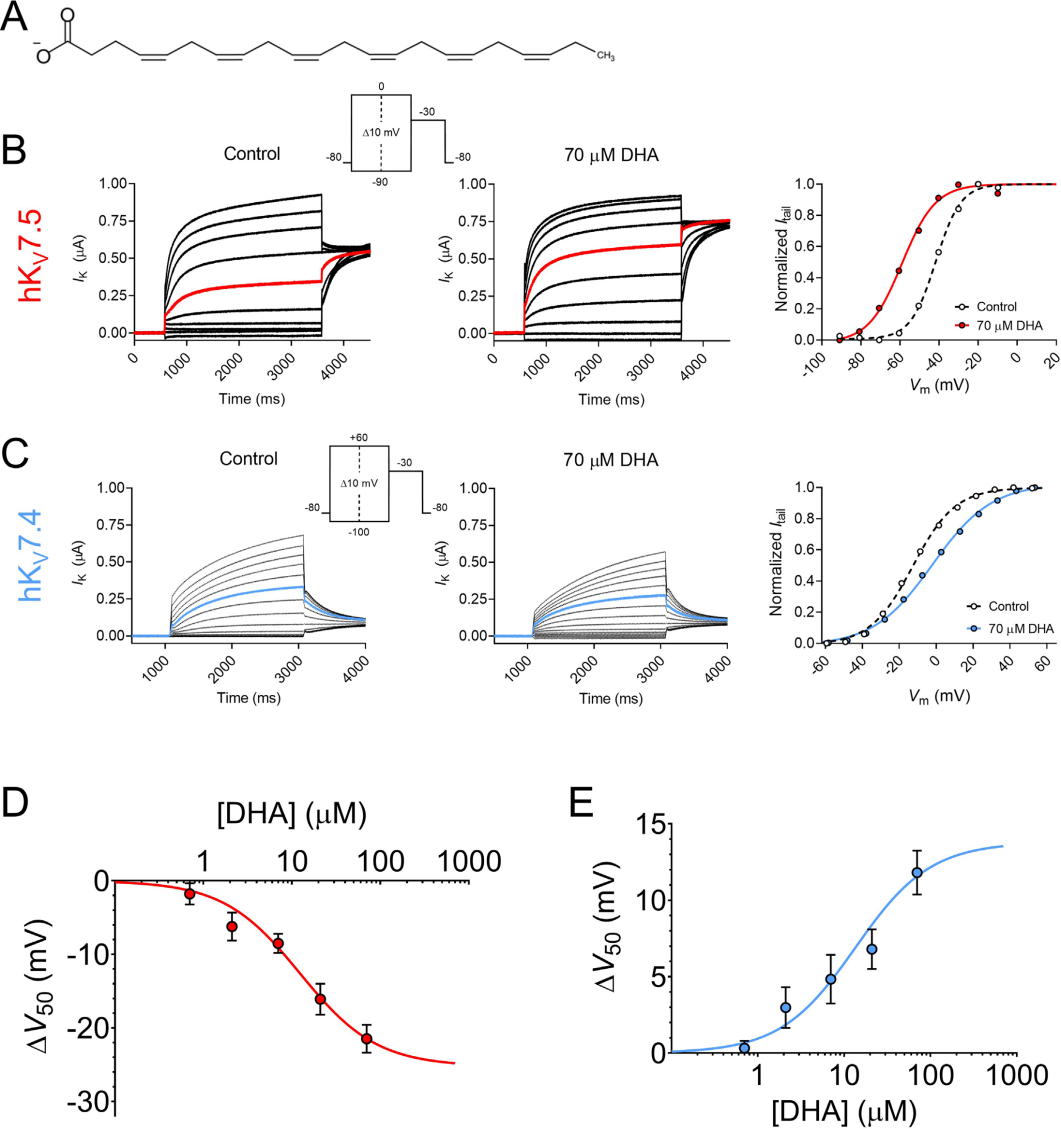
Docosahexaenoic acid facilitates the activation of hK_V_7.5 but inhibits the activation of hK_V_7.4. (**A**) Molecular structure of DHA. (**B**) Representative current family with corresponding G(V) curve of hK_V_7.5 in the absence (left) and presence (middle) of 70 µM DHA. Currents generated by the voltage protocol shown as inset. Red traces denote current generated by a test voltage to –40 mV. The G(V) curves (right) have been normalized between 0 and 1, as described in Materials and methods, to better visualize shifts in V_50_. Curves represent Boltzmann fits (see Materials and methods for details). V_50_ for this specific cell: V_50,ctrl_ = –41.7 mV, V_50,DHA_ = –59 mV. (**C**) Same as in B but for hK_V_7.4. Blue traces denote current generated by a test voltage to 0 mV. V_50_ for this specific cell: V_50,ctrl_ = –12.6 mV, V_50,DHA_ = –2.0 mV. (**D–E**) Concentration-response curve of the DHA effect on V_50_ of hK_V_7.5 (**D**) and hK_V_7.4 (**E**). Curves represent concentration-response fits (see Materials and methods for details). Best fits: ΔV_50,max_ is –25.3 mV for hK_V_7.5 and +13.8 mV for hK_V_7.4. EC_50_ is 12 µM for hK_V_7.5 and 14 µM for hK_V_7.4. Data shown as mean ± SEM. n = 3–15. See also Figure 1—figure supplement 1, Figure 1—source data 1. Figure 1—source data 1.Numerical data for Figure 1.

We began with investigating the effects of the physiologically abundant (Kim et al., 2014) PUFA DHA (molecular structure shown in Figure 1A) on homotetrameric hK_V_7.5 or hK_V_7.4 channels expressed in *Xenopus* oocytes. Activation of the hK_V_7.5 channel was facilitated by 70 μM DHA, as was evident by the significant shift in the midpoint of the voltage dependence of channel opening (V_50_) toward more negative voltages (Figure 1B, average shift of –21.5 ± 1.9 mV, p = <0.0001). This allows hK_V_7.5 channels to open and conduct a K^+^ current at more negative voltages in the presence of DHA. 70 μM DHA did not cause a consistent change in the maximum conductance (G_max_) of the hK_V_7.5 channel (average relative ΔG_max_ was 1.06 ± 0.11, p = 0.59).

**Figure 1—figure supplement 1.**
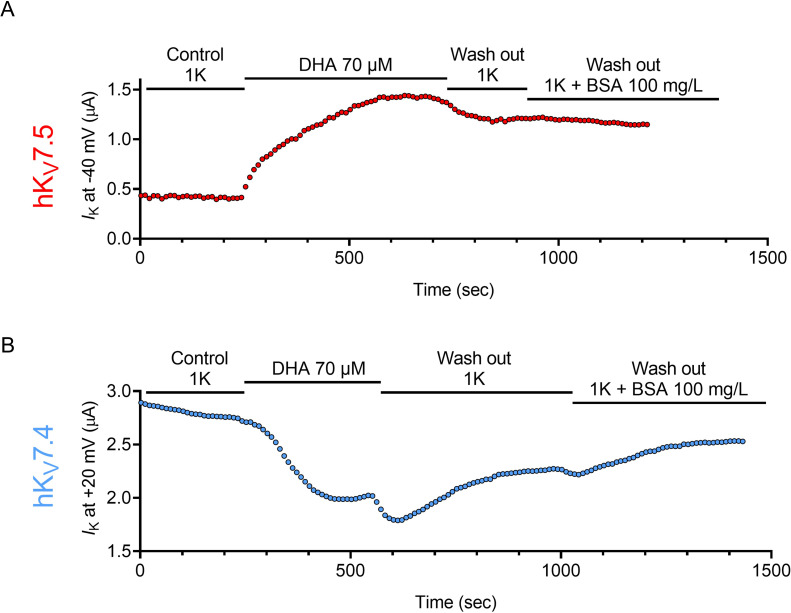
The DHA response has a rapid onset for both hK_V_7.5 and hK_V_7.4. Representative data showing wash-in and wash-out of 70 µM DHA on hK_V_7.5 (**A**) and hK_V_7.4 (**B**) for one oocyte each. Data points represent the current amplitude recorded following a test voltage to –40 mV (for hK_V_7.5) or +20 mV (for hK_V_7.4). The onset of DHA-induced activation of the hK_V_7.5 channel was quick, reaching a stable level of enhanced current within 5 min. Neither standard recording solution nor recording solution supplemented with BSA completely removed the DHA effect, and the enhanced current amplitude remained at almost threefold that of the baseline current amplitude. The onset of DHA-induced inhibition of the hK_V_7.4 channel was also quick, reaching a stable level of reduced current amplitude within 4.5 min. While not fully reversible, the DHA effect was reduced following re-perfusion with standard recording solution. Recording solution supplemented with 100 mg/mL BSA also reduced the inhibitory effect, with the current amplitude reaching approximately 89% of baseline current amplitude. Related to Figure 1.

In clear contrast to the facilitated activation observed for hK_V_7.5, the activation of the hK_V_7.4 channel was inhibited by 70 μM DHA, as was seen by the significant shift in the V_50_ towards more positive voltages (Figure 1C, average shift of +11.8 ± 1.4 mV, p = <0.0001). Furthermore, the application of DHA led to a more shallow slope of the G(V) curve (average slope factors were 13.1 ± 0.2 mV and 17.6 ± 0.5 mV in the absence and presence of 70 μM DHA, respectively). 70 μM DHA did not cause a consistent change in G_max_ of the hK_V_7.4 channel (average relative ΔG_max_ was 1.09 ± 0.08, p = 0.28). Because 70 μM DHA induced significant shifts in V_50_ of both hK_V_7.5 and hK_V_7.4, although in opposite directions, without affecting G_max_ we will throughout the remainder of this study focus our analysis of PUFA effects on V_50_, and G(V) curves shown in figures reporting on PUFA effects will be normalized to visually emphasize V_50_ shifts.

The DHA-evoked shift in the V_50_ of hK_V_7.5 was significant at concentrations as low as 7 μM (Figure 1D, ∆V_50_ = –8.5 ± 1.3 mV, p = 0.0002). The concentration-response curve for the DHA effect on hK_V_7.5 predicts a maximum V_50_ shift of –25.3 mV, with 12 μM required for 50% of the maximum shift (EC_50_ = 12 μM). The DHA effect on the V_50_ of hK_V_7.4 was also significant at 7 μM (Figure 1E, ∆V_50_ = +4.8 ± 1.6 mV, p = 0.01). The concentration-response curve for the DHA effect on hK_V_7.4 predicts a maximum V_50_ shift of +13.8 mV, with an EC_50_ of 14 μM. The onset of the DHA effect was relatively fast for both the hK_V_7.5 and hK_V_7.4 channels, reaching a stable level within about 6 min of application (Figure 1—figure supplement 1). The DHA effect proved difficult to wash out or reverse, as re-perfusion with control solution or control solution supplemented with 100 mg/mL bovine serum albumin (BSA) only partially restored baseline current amplitude for hK_V_7.5 and hK_V_7.4 (Figure 1—figure supplement 1). Altogether, the PUFA DHA induces a concentration-dependent *facilitation* of activation of the hK_V_7.5 channel, and a concentration-dependent *inhibition* of activation of the hK_V_7.4 channel.

### The hK_V_7.5 response, but not the hK_V_7.4 response, changes direction in an electrostatic manner

To further understand the molecular basis of the DHA response, we examined the importance of the charge of the DHA head group. Several previous studies identify electrostatic interactions between positively charged arginines in the upper half of S4 of the VSD and the negatively charged head group on PUFAs (and their analogues) as fundamental for facilitating the activation of hK_V_7.1, hK_V_7.2/7.3 and some other K_V_ channels (Börjesson and Elinder, 2011; Liin et al., 2015; Liin et al., 2016; Liin et al., 2018; Martín et al., 2021). This is seen as a shift in V_50_ toward more negative voltages. The electrostatic PUFA effect on V_50_ can be tuned, from inducing negative to positive shifts in V_50_, by altering the charge of the PUFA head group (Börjesson et al., 2010; Liin et al., 2015). To investigate if the same electrostatic mechanism is at play in the responses of hK_V_7.5 and hK_V_7.4, we compared the DHA response of the channels with: (1) a DHA analogue with an uncharged methyl ester head group (DHA-me), and (2) a DHA analogue with a positively charged amine head group (DHA+).

**Figure 2.**
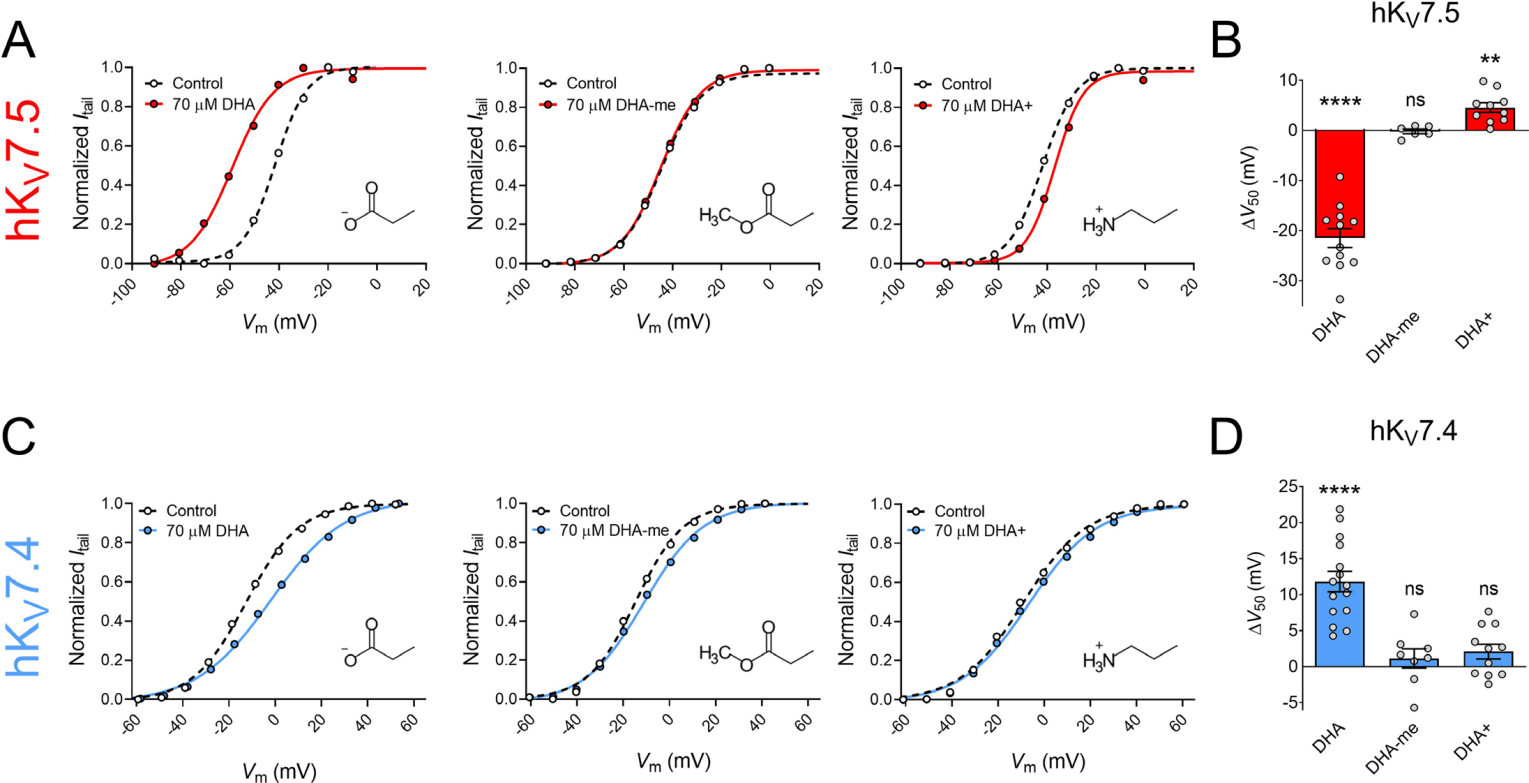
The impact of PUFA head group charge for hK_V_7.5 and hK_V_7.4 effects. (**A–B**) Impact of the head group charge on the ability of DHA to shift V_50_ of hK_V_7.5, assessed by comparing the effect of negatively charged DHA, uncharged DHA-me, and positively charged DHA+ at 70 µM. (**A**) Representative examples of the negative shift in V_50_ induced by DHA (left, same data as shown in Figure 1B), the lack of effect of DHA-me (middle, V_50,ctrl_ = –43.9 mV; V_50,DHA-me_ = -44.6 mV), and the positive shift in V_50_ induced by DHA+ (right, V_50,ctrl_ = –42.0 mV; V_50,DHA+_ = –37.0 mV). Molecular structure of head groups are shown as insets. (**B**) Summary of responses to indicated DHA compound. Data shown as mean ± SEM. n = 6–12. Statistics denote one sample *t* test against a hypothetical mean of 0 mV. ns denotes not significant, ** denotes p ≤ 0.01, **** denotes p ≤ 0.0001. (**C–D**) same as in A-B but for hK_V_7.4. (**C**) Representative examples of the depolarizing shift in V_50_ induced by DHA (left, same data as shown in Figure 1C), the lack of effect of DHA-me (middle, V_50,ctrl_ = –14.6 mV; V_50,DHA-me_ = –11.1 mV), and the lack of effect of DHA+ (right, V_50,ctrl_ = –9.1 mV; V_50,DHA+_ = –6.6 mV). (**D**) Summary of responses to indicated DHA compound. Data shown as mean ± SEM. n = 8–15. Statistics denote one sample *t* test against a hypothetical mean of 0 mV. ns denotes not significant, **** denotes p ≤ 0.0001. See also Figure 2—source data 1. Figure 2—source data 1.Numerical data for Figure 2.

70 μM of DHA-me did not significantly shift V_50_ of hK_V_7.5 (Figure 2A, ∆V_50_ = –0.2 ± 0.5 mV, p = 0.67). In contrast, 70 μM DHA+ brought on a small, but significant positive shift in V_50_ of hK_V_7.5 (Figure 2A, ΔV_50_ = +4.5 ± 0.9 mV, p = 0.001). Thus, for hK_V_7.5 the negatively charged DHA facilitates activation, the uncharged DHA-me has no effect, and the positively charged DHA+ inhibits channel activation (effects summarized in bar graph of Figure 2B). This is in line with the lipoelectric mechanism that has been proposed to explain PUFA effects on the hK_V_7.1 and hK_V_7.2/7.3 channels (Liin et al., 2015; Liin et al., 2016).

Neither 70 μM of DHA-me nor 70 μM of DHA+ had significant effects on V_50_ of hK_V_7.4 (Figure 2C, ΔV_50_ = +1.1 ± 1.3 mV, p = 0.42 for DHA-me; ∆V_50_ = +2.1 ± 1.0 mV, p = 0.068 for DHA+). Thus, while the negatively charged DHA caused a positive shift in V_50_ of hK_V_7.4, neither the uncharged DHA-me nor the positively charged DHA+ altered the V_50_ of hK_V_7.4 (effects summarized in bar graph of Figure 2D). Even though a negative charge of the head group seems to be important for the effect, the responses are not in line with the lipoelectric mechanism, making hK_V_7.4 unique among the hK_V_7 channels.

### Both hK_V_7.5 and hK_V_7.4 respond broadly to PUFAs, although with varied magnitudes of responses

**Figure 3.**
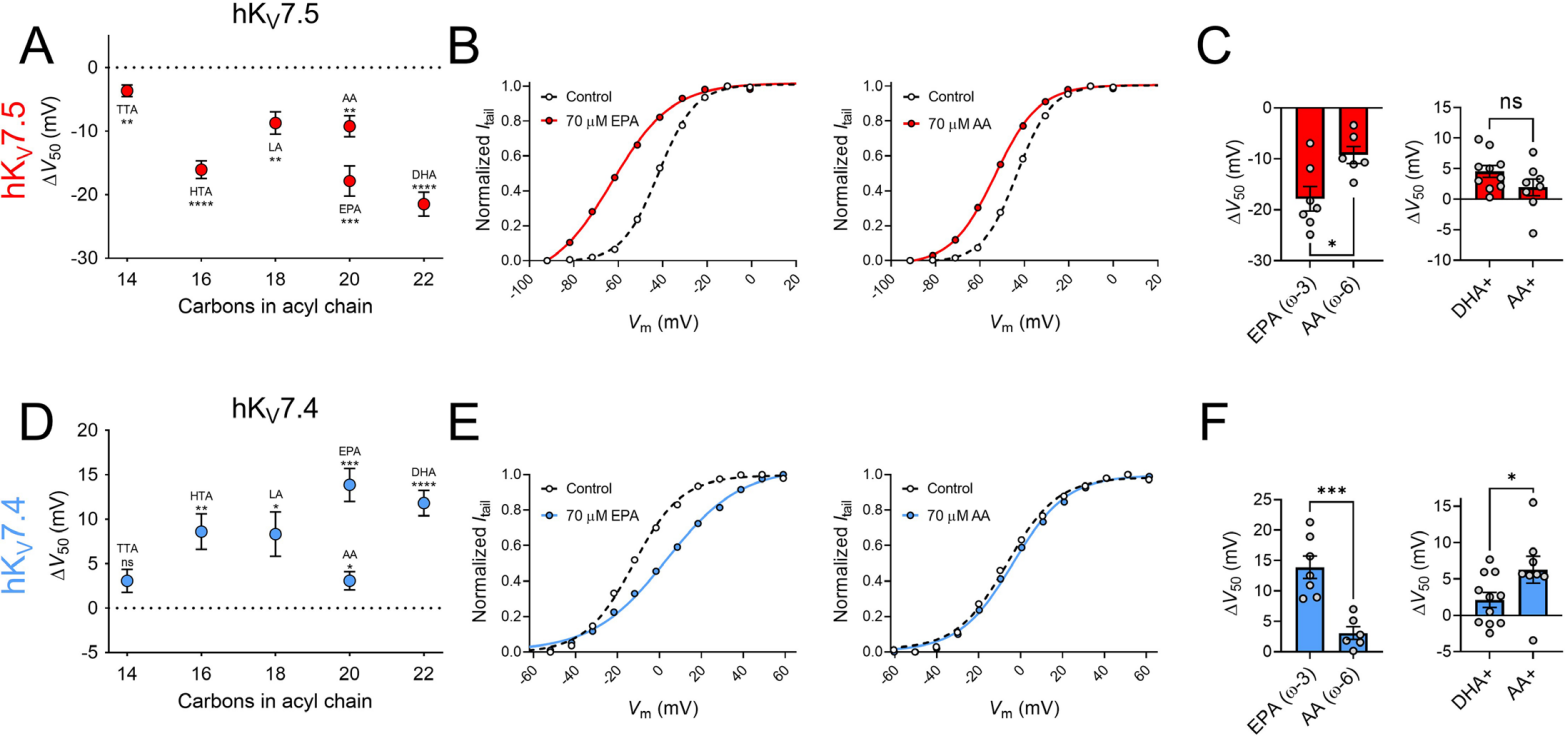
The impact of PUFA tail properties for hK_V_7.5 and hK_V_7.4 effects. (**A–C**) Impact of PUFA tail properties for the ability of PUFAs to shift V_50_ of hK_V_7.5, assessed by comparing the response to 70 µM of indicated PUFAs (see Table 1 for molecular structures). (**A**) Average effect of indicated PUFAs. Data shown as mean ± SEM. n = 6–8. Statistics denote one sample *t* test against a hypothetical mean of 0 mV ns denotes not significant, ** denotes p ≤ 0.01, *** denotes p ≤ 0.001, **** denotes p ≤ 0.0001. (**B**) Representative examples of response to 70 µM of the ω–3 PUFA EPA (left, V_50,ctrl_ = -41.9 mV; V_50,EPA_ = -62.8 mV) and ω–6 PUFA AA (middle, V_50,ctrl_ = –43.3 mV; V_50,AA_ = –53.1 mV). (**C**) Bar graphs with comparison of average response to 70 µM of EPA and AA (n = 6–7) and DHA+ and AA+ (n = 8–10). Statistics denote Student’s *t* test. ns denotes not significant, * denotes p ≤ 0.05. (**D–F**) same as in A-C but for hK_V_7.4. (**D**) n = 6–8. (**E**) Left: V_50,ctrl_ = –12.3 mV; V_50,EPA_ = +3.4 mV. Middle: V_50,ctrl_ = –6.5 mV; V_50,AA_ = –3.6 mV. (**F**) n = 6–7 and n = 8–11, respectively. * denotes p ≤ 0.05, ** denotes p ≤ 0.01, *** denotes p ≤ 0.001, **** denotes p ≤ 0.0001. See also Figure 3—source data 1. Figure 3—source data 1.Numerical data for Figure 3.

Next, we studied if the effects on hK_V_7.5 and hK_V_7.4 are specific to DHA or if they are shared among different PUFAs by testing a series of PUFAs, listed in Table 1. These PUFAs vary in their molecular properties, such as the length of the tail and the number and position of double bonds in the tail. Figure 3A shows a plot of the magnitude of ΔV_50_ following exposure of hK_V_7.5 to PUFAs (at 70 μM) against the number of carbon atoms in each respective PUFA tail. All PUFAs, regardless of length (14–22 carbon atoms) significantly shifted V_50_ of hK_V_7.5 toward more negative voltages, although to different extents. The magnitude of the PUFA-induced ΔV_50_ of hK_V_7.5 followed a pattern of TTA < LA = AA < HTA ≤ EPA < DHA. One observation is that the ω–6 PUFAs LA and AA induced smaller shifts than the ω–3 PUFAs HTA, EPA and DHA. Both AA and EPA are 20 carbon atoms long. However, while AA is an ω–6 PUFA, EPA is an ω–3 PUFA (Table 1). Figure 3B shows representative G(V) curves for hK_V_7.5 that highlight the larger shift in V_50_ evoked by 70 μM of EPA (left) than by 70 μM of AA (right). On average, EPA induced a shift of –17.9 ± 2.4 mV, which was significantly larger than the shift induced by AA (ΔV_50_ = –9.3 ± 1.7 mV; Figure 3C; p = 0.015). These results suggest that in PUFAs of equal length, the ω-number has an impact on the magnitude of the PUFA response of hK_V_7.5. We also compared the positively charged amine analogue DHA+ to an amine analogue of AA (AA+). The positive shift in V_50_ of hK_V_7.5 by 70 μM AA+ was slightly smaller than that observed with DHA+, and did not differ significantly from a hypothetical shift of 0 mV (Figure 3C, AA+ ΔV_50_ = +1.9 ± 1.4 mV, p = 0.2). However, there was also no statistically significant difference between the effects induced by DHA+ and AA+ (p = 0.13), which indicates that the importance of the ω-number for the amine analogues should be interpreted with caution. Altogether, these experiments show that many PUFAs activate the hK_V_7.5 channel and suggest that the hK_V_7.5 channel shows a preference toward ω–3 over ω–6 PUFAs.

**Table 1.** List of PUFAs and PUFA analogues used in this study.

IUPAC name	Lipid no. (C:DBs)	Omega no.	Molecular structure
**PUFAs:**			
Tetradecatrienoic acid (TTA)	14:3	ω–3	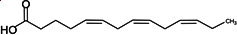
Hexadecatrienoic acid (HTA)	16:3	ω–3	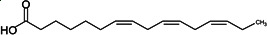
Linoleic acid (LA)	18:2	ω–6	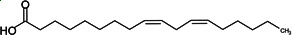
Arachidonic acid (AA)	20:4	ω–6	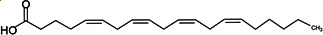
Eicosapentaenoic acid (EPA)	20:5	ω–3	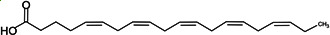
Docosahexaenoic acid (DHA)	22:6	ω–3	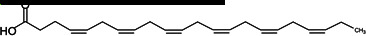
**PUFA analogues:**			
Arachidonoyl amine (AA+)	20:4	ω–6	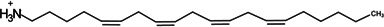
Docosahexaneoic acid methyl ester (DHA-me)	22:6	ω–3	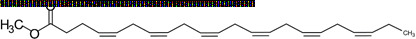
Docosahexaenoyl amine (DHA+)	22:6	ω–3	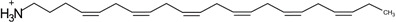

C denotes number of carbon atoms in tail, DB denotes double bonds.

Figure 3D shows a plot of the magnitude of ΔV_50_ following exposure to PUFAs (at 70 μM) against the number of carbon atoms in each respective PUFA tail for the hK_V_7.4 channel. The shortest PUFA, TTA, did not evoke a significant shift in V_50_ (ΔV_50_ = +3.1 ± 1.3 mV, p = 0.051). All remaining PUFAs (at a concentration of 70 μM), however, significantly shifted V_50_ of hK_V_7.4 toward positive voltages. The magnitude of the PUFA-induced ΔV_50_ of hK_V_7.4 followed a pattern of TTA <AA < LA ≤ HTA < DHA < EPA with no clear pattern in magnitude of effect based on the ω-number of the PUFA tail. Although the ω–3 PUFA EPA induced a larger shift than the ω–6 PUFA AA (Figure 3E, F, EPA ΔV_50_ = +13.9 ± 1.9 mV; AA ΔV_50_ = +3.1 ± 1.0 mV, p = <0.001), AA+ caused a larger shift in V_50_ compared to DHA+ (Figure 3F, DHA+ ΔV_50_ = +2.1 ± 1.0 mV; AA+ ΔV_50_ = +6.3 ± 1.8 mV, p = 0.049). Moreover, the ω–6 PUFA LA induced comparable shifts in V_50_ to those of the ω–3 PUFA HTA. Altogether, these experiments show that many PUFAs inhibit the hK_V_7.4 channel, with no obvious pattern of which PUFAs hK_V_7.4 shows a preference toward.

### hK_V_7.4/7.5 co-expression and disease-associated hK_V_7.4 mutations, but not hKCNE4 co-expression, influence the response to DHA

**Figure 4.**
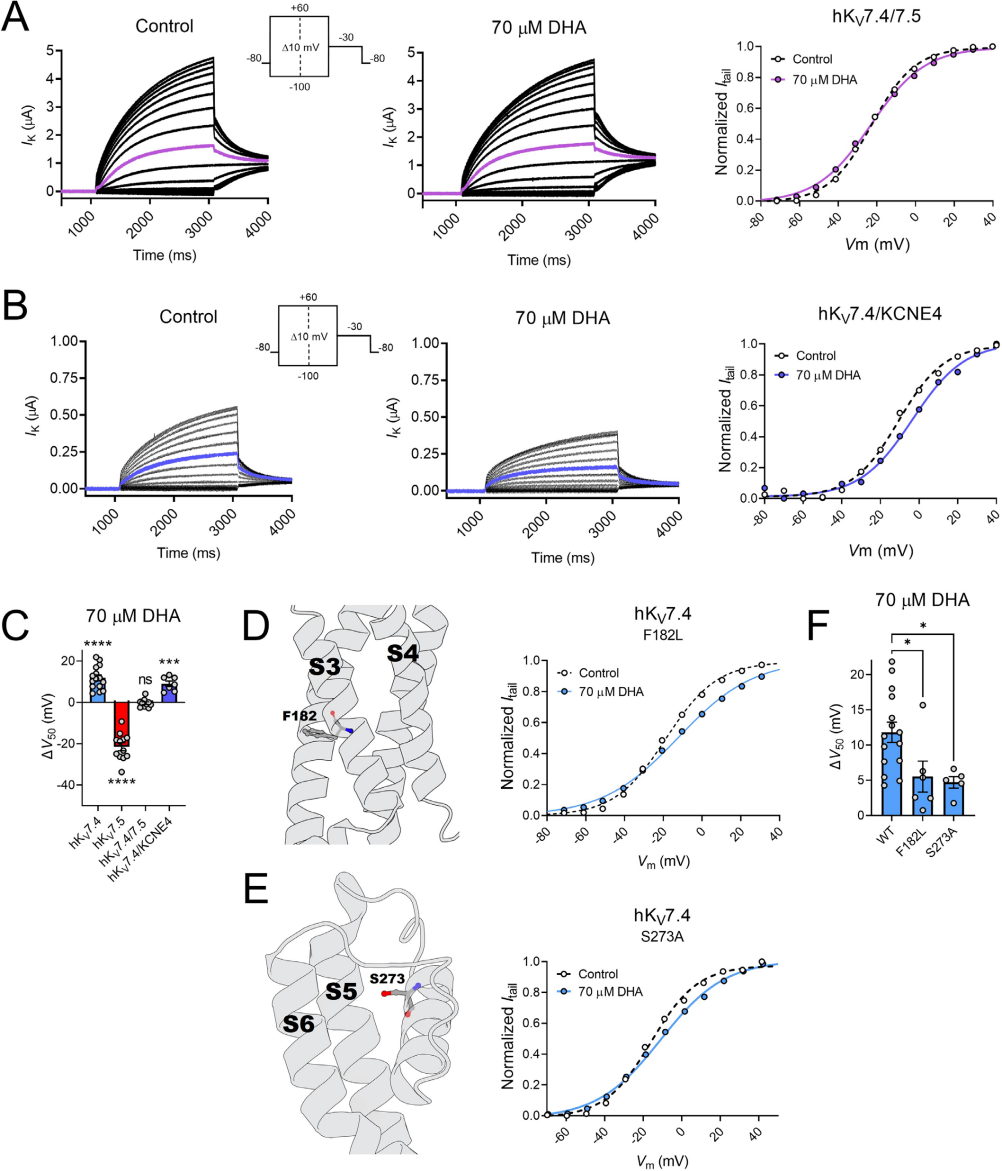
hK_V_7.4/7.5 co-expression and disease-associated hKv7.4 mutations, but not hKCNE4 co-expression, influence the response to DHA. (**A**) Representative current family with corresponding G(V) curve of hK_V_7.4/7.5 in the absence (left) and presence (middle) of 70 µM DHA. Purple traces denote current generated by a test voltage to –20 mV. Curves represent Boltzmann fits. V_50_ for this specific cell: V_50,ctrl_ = –22.8 mV; V_50,DHA_ = –23.1 mV. (**B**) Representative current family with corresponding G(V) curve of hK_V_7.4/KCNE4 in the absence (left) and presence (middle) of 70 µM DHA. Blue traces denote current generated by a test voltage to 0 mV. Curves represent Boltzmann fits. V_50_ for this specific cell: V_50,ctrl_ = –10.7 mV; V_50,DHA_ = –3.5 mV. (**C**) Summary of response of hK_V_7.4/7.5 and hK_V_7.4/KCNE4 to 70 µM DHA, with responses of hK_V_7.4 and hK_V_7.5 for reference. Data shown as mean ± SEM. n = 7–15. Statistics denote one sample *t* test against a hypothetical mean of 0 mV. ns denotes not significant, *** denotes p ≤ 0.001, **** denotes p ≤ 0.0001. (**D–E**) Impact of hK_V_7.4 mutations F182L (**D**) and S273A (**E**) on the response to DHA. Structural model (PDB ID - 7BYL, Li et al., 2021) of hK_V_7.4 with position of F182 and S273 marked. Representative G(V) curve of indicated hK_V_7.4 mutants in the absence and presence of 70 µM DHA. Curves represent Boltzmann fits. For these specific cells: F182L: V_50,ctrl_ = –18.7 mV; V_50,DHA_ = –12.4 mV. For S273A: V_50,ctrl_ = –15.8 mV; V_50,DHA_ = –10.8 mV. (**F**) Summary of response of WT hK_V_7.4 and indicated mutants to 70 µM DHA. Data shown as mean ± SEM. n = 5–15. Statistics denote one-way ANOVA followed by Dunnett’s multiple comparisons test to compare the response of mutants to that of the wild-type. * denotes p ≤ 0.05. See also Figure 4—source data 1. Figure 4—source data 1.Numerical data for Figure 4.

We next characterized the DHA response of oocytes co-injected with cRNAs encoding hK_V_7.4 and hK_V_7.5 (referred to as hK_V_7.4/7.5) to allow for the potential formation of heteromeric channels containing hK_V_7.4 and hK_V_7.5 subunits. Oocytes co-injected with hK_V_7.4 and hK_V_7.5 generated currents with biophysical properties that fell between those of each homomeric channel complex (hK_V_7.4 V_50_ = –11.1 ± 1.4 mV; hK_V_7.5 V_50_ = –42.2 ± 0.7 mV; hK_V_7.4/7.5 V_50_ = −22.9 ± 0.6 mV, Table 2). This is in agreement with previous studies showing a V_50_ of co-expressed hK_V_7.4/7.5 channels intermediate to that of homomeric channels (Brueggemann et al., 2011). In addition, 70 μM of DHA induced an effect on hK_V_7.4/7.5 intermediate to that of homomeric hK_V_7.4 and hK_V_7.5 channels, with no change in V_50_ (Figure 4A, C, ΔV_50_ = –0.4 ± 0.6 mV, p = 0.53).

**Table 2.** Biophysical properties of tested constructs under control conditions. Table 2—source data 1.Numerical data for Table 2.

Channel	Variant	V_50_ (mV)Mean ± SEM	Slope (mV)Mean ± SEM	n
hK_V_7.4	Wild-type	–11.1 ± 1.4	13.1 ± 0.2	20
	F182L	–21.0 ± 2.9	12.0 ± 0.5	10
	R213Q	4.6 ± 1.3	12.2 ± 0.4	24
	R216Q	–26 ± 0.8	11.4 ± 0.3	24
	R219Q	13.3 ± 1.0	12.7 ± 0.2	24
	S273A	–15.9 ± 3.1	14.1 ± 1.9	15
hK_V_7.5	Wild-type	–42.2 ± 0.7	8.4 ± 0.2	23
hK_V_7.4/7.5	Wild-type	–22.9 ± 0.6	10.8 ± 0.2	18
hK_V_7.4/KCNE4	Wild-type	–12.9 ± 2.2	12.9 ± 1.3	7

n denotes number of cells. V_50_ and slope were determined from Boltzmann fits, as described in Materials and methods. See also Table 2—source data 1.

K_V_7.4 has been shown to co-assemble with the KCNE4 subunit in, for instance, vascular smooth muscle tissue (Jepps et al., 2015). We therefore characterized the DHA response of oocytes co-injected with cRNAs encoding hK_V_7.4 and hKCNE4 (referred to as hK_V_7.4/KCNE4). In agreement with previous studies (Vanoye et al., 2009; Jepps et al., 2015), this generated currents with biophysical properties fairly similar to those of hK_V_7.4 alone (Table 2). 70 μM of DHA shifted V_50_ of hK_V_7.4/KCNE4 toward more positive voltages (Figure 4B–C, ΔV_50_ = +8.9 ± 1.3 mV, p = 0.0005). This effect was comparable to that on hK_V_7.4 alone (p = 0.23), suggesting the hKCNE4 subunit does not alter the DHA response of the hK_V_7.4 channel.

Several mutations in the *KCNQ4* gene (encoding hK_V_7.4) have been identified in humans and are often linked to DFNA2 non-syndromic hearing loss (Jung et al., 2019; Rim et al., 2021), although the possible contribution to pathology remains to be determined for many mutations. Notably, the F182L and S273A missense mutations in hK_V_7.4 (Figure 4D–E), which are suspected to be linked to impaired hearing (Jung et al., 2019; Kim et al., 2011), exchanges the native hK_V_7.4 residue for the hK_V_7.5 counterpart. By means of site-directed mutagenesis, we substituted these residues in hK_V_7.4, one by one. Under control conditions, we found both mutants to behave fairly similar to wild-type hK_V_7.4 (Table 2). The S273A mutant had a V_50_ comparable to wild-type hK_V_7.4, whereas the V_50_ of the F182L mutant was shifted about 10 mV toward more negative voltages compared to wild-type hK_V_7.4 (Table 2). However, both mutations impaired the inhibitory hK_V_7.4 response to DHA. 70 µM of DHA shifted V_50_ of the F182L mutant by only +5.5 ± 2.2 mV and the S273A mutant by +4.7 ± 0.8 mV (Figure 4D–F).

Altogether, these experiments suggest that co-expression of hK_V_7.4 and hK_V_7.5 subunits, or substitution of specific residues in hK_V_7.4 to the hK_V_7.5 counterpart, alter the DHA response of hK_V_7.4 to approach that of hK_V_7.5. However, hK_V_7.4 co-expression with the hKCNE4 auxiliary subunit did not alter the DHA response.

### The DHA response of hK_V_7.5 is greater than that of other hK_V_7 subtypes

**Figure 5.**
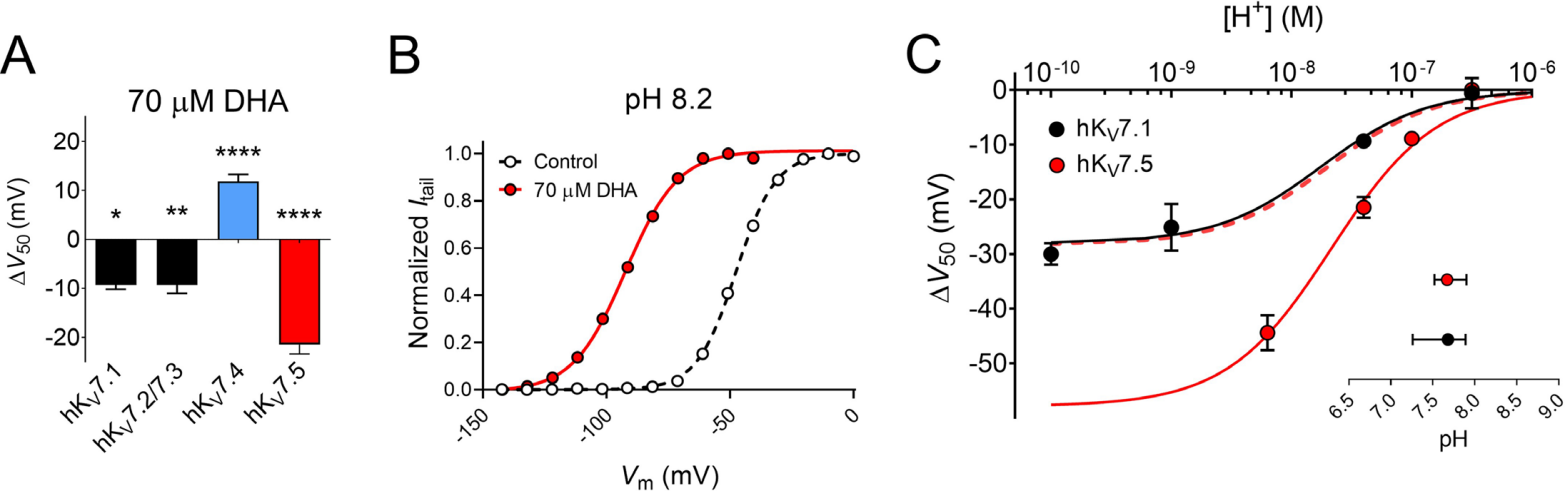
Extracellular pH tunes the DHA response of hK_V_7.5. (**A**) Comparison of the V_50_ shift of hK_V_7 subtypes in response to 70 µM DHA. Data for hK_V_7.1 and hK_V_7.2/7.3 shown as reported in Liin et al., 2016; Liin et al., 2015. Data for hK_V_7.4 and hK_V_7.5 from the present study. Data shown as mean ± SEM. n = 3–15. Statistics denote one sample *t* test against a hypothetical mean of 0 mV. * denotes p ≤ 0.05, ** denotes p ≤ 0.01, **** denotes p ≤ 0.0001. (**B–C**) Altering extracellular pH tunes the shift in V_50_ of hK_V_7.5 induced by 70 µM DHA, with a greater response observed at more alkaline pH. Note that in all experiments, the pH of the control solution and DHA-supplemented control solution was identical. (**B**) Representative example of the DHA response at pH 8.2 (V_50,ctrl_ = –47.6 mV; V_50,DHA_ = –92.4 mV). (**C**) pH-response curve for the DHA effect on V_50_. Data shown as mean ± SEM. n = 3–9. Curves represent pH-response fits (see Materials and methods for details). Best fit for hK_V_7.5: ΔV_50,max_ is –57.9 mV. Data for hK_V_7.1 as reported in Liin et al., 2015 is included for comparison (Best fit for hK_V_7.1: ΔV_50,max_ is –26.3 mV.) Dashed line denotes the pH-response curve for hK_V_7.5 normalized to ΔV_50,max_ for hK_V_7.1 to illustrate the comparable apparent pKa. Inset shows apparent pKa with 95% CI: hK_V_7.1 apparent pKa = 7.68 with 95% CI [7.26, 7.89]; hK_V_7.5 apparent pKa = 7.67 with 95% CI [7.52, 7.90]. Note that inconsistent behavior of hK_V_7.5 under control conditions at pH higher than 8.2 prevented us from determining the DHA effect on hK_V_7.5 at pH 9 and 10. See also Figure 5—figure supplement 1, Figure 5—source data 1. Figure 5—source data 1.Numerical data for Figure 5.

We have in previous studies shown that 70 μM of DHA shifts the V_50_ of the hK_V_7.1 and hK_V_7.2/7.3 channels by about –9 mV (Figure 5A; hK_V_7.1 ΔV_50_ = –9.3 ± 0.9 mV, as reported in Liin et al., 2015; hK_V_7.2/7.3 ΔV_50_ = –9.3 ± 1.7 mV, as reported in Liin et al., 2016). Thus, the extent of the hK_V_7.5 shift induced by 70 μM DHA is greater than that of hK_V_7.1 or hK_V_7.2/7.3 (Figure 5A). Our experiments indicated the necessity of a negatively charged DHA head group to elicit the activating effect on hK_V_7.5 (see Figure 2A). A negatively charged head group is promoted by alkaline pH, which triggers proton dissociation (Börjesson et al., 2008; Hamilton, 1998). In a previous study, the apparent pKa of DHA when near hK_V_7.1 (i.e. the pH at which 50% of the maximal DHA effect is seen, interpreted as the pH at which 50% of the DHA molecules in a lipid environment are negatively charged) was determined to be pH 7.7 (Liin et al., 2015). One possible underlying cause of the larger DHA effect on hK_V_7.5 is that the local pH environment at hK_V_7.5 promotes DHA deprotonation (i.e. inducing a lower apparent pKa of DHA), thus rendering a greater fraction of the DHA molecules negatively charged and capable of activating the channel. To test this, we assessed the effect of 70 μM DHA on hK_V_7.5 with the extracellular pH adjusted to either more alkaline (pH = 8.2) or acidic (pH = 6.5, or 7.0) values. Adjusting the extracellular pH had only minor effects on V_50_ under control conditions (Figure 5—figure supplement 1). In contrast, the magnitude of the DHA-induced shift varied with pH. At pH 8.2, at which a majority of DHA molecules are expected to be deprotonated, DHA shifts of V_50_ were almost two-fold greater than at physiological pH (pH 7.4 ΔV_50_ = –21.5 ± 1.9 mV; pH 8.2 ΔV_50_ = –44.4 ± 3.2 mV, p < 0.0001, Student’s *t* test, representative example in Figure 5B). The magnitude of the DHA effect was reduced as the pH was gradually titrated toward more acidic pH (Figure 5C; Figure 5—figure supplement 1), with no shift in V_50_ by 70 μM DHA observed at pH 6.5 (ΔV_50_ = +0.1 ± 1.0 mV, p = 0.96). The pH dependence of ΔV_50_ induced by DHA for the hK_V_7.1 channel is also plotted in Figure 5C, to allow for comparison between the two hK_V_7 subtypes. While there is a clear difference in the extrapolated maximum shifts for the two channels (hK_V_7.1 ΔV_50,max_ = –26.3 mV with 95% CI [-18.4,–34.3]; hK_V_7.5 ΔV_50,max_ = –57.9 mV with 95% CI [-47.6,–68.2]), the pH required to induce 50% of the maximum ΔV_50_ (i.e. the apparent pKa values) are similar (inset of Figure 5C, hK_V_7.1 apparent pKa = 7.68 with 95% CI [7.26, 7.89]; hK_V_7.5 apparent pKa = 7.67 with 95% CI [7.52, 7.90]). This indicates that the difference in the DHA effect between hK_V_7.1 and hK_V_7.5 is a question of increased magnitude, rather than a matter of different apparent pKa of DHA at the channels.

**Figure 5—figure supplement 1.**
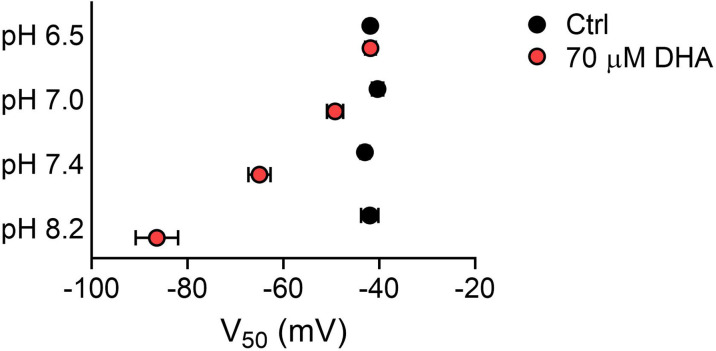
pH dependence of the DHA response of hK_V_7.5. Altering extracellular pH does not affect V_50_ of hK_V_7.5 under control conditions (black symbols), which remains close to –40 mV in experiments using control recording solution with the indicated pH values. In contrast, altering extracellular pH tunes the shift in V_50_ of hK_V_7.5 induced by 70 µM DHA, with a greater response observed at more alkaline pH. Red symbols denote V_50_ in experiments using DHA-supplemented recording solution with indicated pH values, which ranges from about –40 mV at pH 6.5 to about –85 mV at pH 8.2. Data shown as mean ± SEM. n = 6–11. The DHA-induced shift in V_50_ (ΔV_50_) shown in Figure 5C is calculated as the difference in V_50_ in control recording solution and DHA-supplemented recording solution at each pH (i.e. the difference between the black and red symbol at each pH), and plotted as a function of H^+^ concentration of the extracellular recording solution. Related to Figure 5.

### Molecular dynamics simulations predict an unconventional inner VSD PUFA site in hK_V_7.4

**Figure 6.**
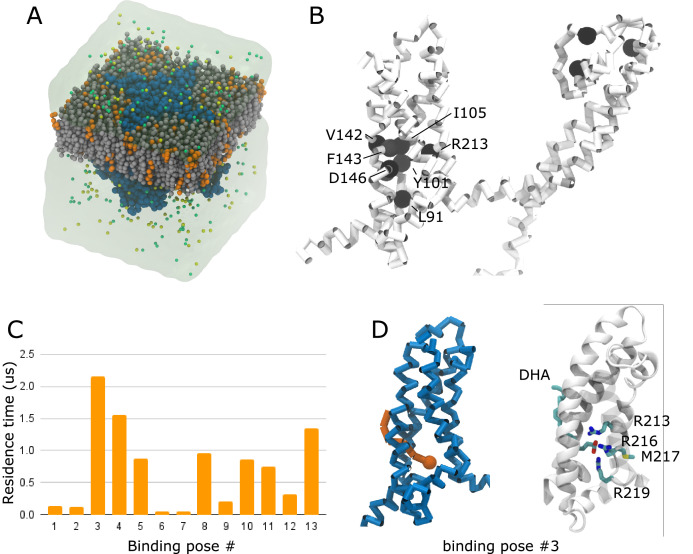
Molecular dynamics simulation predicts an unconventional inner VSD site for DHA in hK_V_7.4. (**A**) Coarse-grained simulation system, containing hK_V_7.4 (blue) in a bilayer made of 75% POPC (grey) and 25% DHA (orange) plunged in a KCl bath. (**B**) Residence times of interactions between DHA and individual hK_V_7.4 residues projected as a color scale ranging from white (short residence times) to black (long residence times). Residues with a residence time above 4.4 µs are highlighted as black spheres. Note the cluster of such residues in the lower half of the VSD. (**C**) Residence time of DHA in 13 different binding poses identified through a clustering analysis. (**D**) The binding pose in the cluster with the highest residence time (cluster #3) is shown in the coarse-grained representation used to run the simulations (left - hK_V_7.4 VSD in blue, DHA in orange) and in an all-atom representation obtained by backmapping the coarse-grained system to all-atom representation (right – protein backbone in white ribbons, DHA and residues interacting with the charged head group as sticks colored by atom type, C – cyan, N – blue, O – red, S – yellow, H omitted for clarity). See also Figure 6—figure supplements 1–3. Source data, including code, for molecular dynamics simulations is accessible at https://osf.io/6fuqs/.

We next turned our attention toward structural components of the hK_V_7.4 channel that may contribute to its unusual PUFA response. In order to determine the interaction sites for DHA on hK_V_7.4, we performed coarse-grained molecular dynamics simulations of the channel, solved in an intermediate activation state, embedded in a membrane consisting of 75% POPC and 25% negatively charged DHA randomly distributed in both membrane leaflets (Figure 6A). Analysis of the residence time of DHA showed overall prolonged interactions with residues on S1 (L91, Y101, and I105), on S2 (V142, F143, and D146), on S4 (R213) and at the top of the pore region (Figure 6B and Figure 6—figure supplement 1A). Out of the 13 interaction sites characterized by density-based clustering (Figure 6—figure supplement 1B), the site at which DHA interacted with the channel the longest (2.156 µs, cluster #3) was comprised of residues located on the lower half of S4. This site was also occupied ~87% of the time (Figure 6C, Figure 6—figure supplement 1B), suggesting favorable interactions between the side chains of R213, R216, and R219 and the head group of DHA, as well as interactions with the backbone of M217. In the representative binding pose of that site, the head group of DHA is bound in the lumen of the lower portion of the VSD, close to the S4 helix (Figure 6D). We will refer to this as an unconventional ‘inner VSD site’, in contrast to the conventional ‘outer VSD site’ described previously for K_V_7.1 (Yazdi et al., 2021). Because of its negative charge, DHA in the inner VSD site presumably prevents the outward motion of the positively charged S4 helix, rationalizing its inhibitory effect. The wedging of the tail between S1 and S2 suggests that the DHA head group accesses this interaction site via this lipid-exposed interface. Repeating these simulations with uncharged DHA changes the interaction pattern drastically. Indeed, neutral DHA shows a high propensity to localize close to the lower half of the S5 and S6 helix, at a considerable distance from the VSD (Figure 6—figure supplement 2). This site presumably has little functional effect, providing an explanation for the lack of effect of the uncharged DHA-me on hK_V_7.4 activation.

**Figure 6—figure supplement 1.**
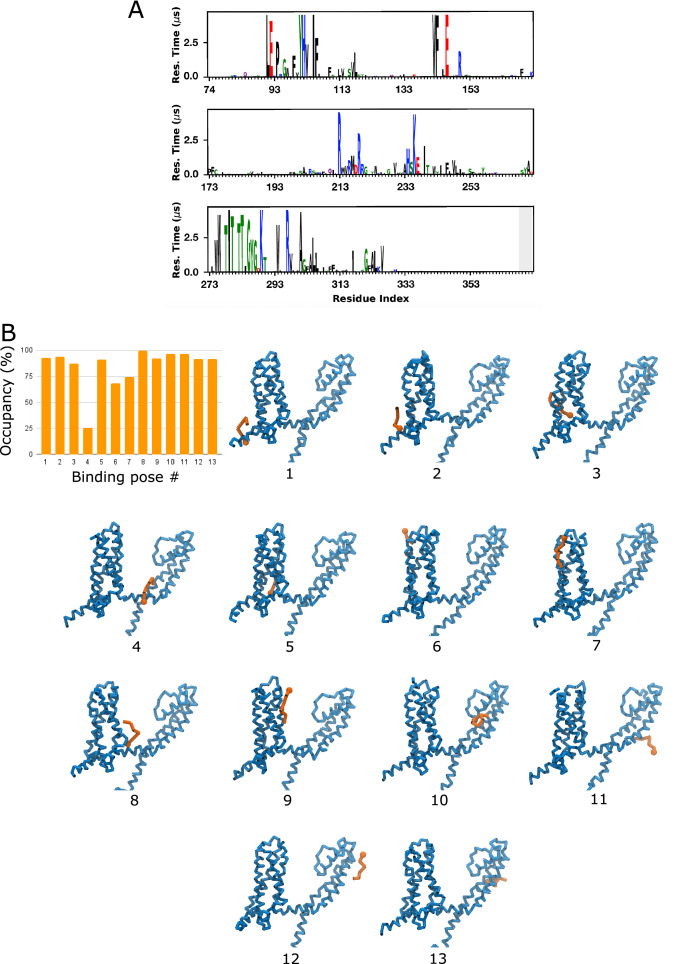
Overview of predicted interaction of negatively charged DHA with hK_V_7.4. (**A**) Residence time of DHA projected along the hK_V_7.4 amino acid sequence, calculated from coarse-grained simulations of hK_V_7.4 embedded in a mixture of POPC and charged DHA. The higher the logo letter, the higher the residence time. (**B**) Occupancy of DHA in 13 different binding poses identified through a clustering analysis and molecular models corresponding to the 13 poses. Related to Figure 6.

**Figure 6—figure supplement 2.**
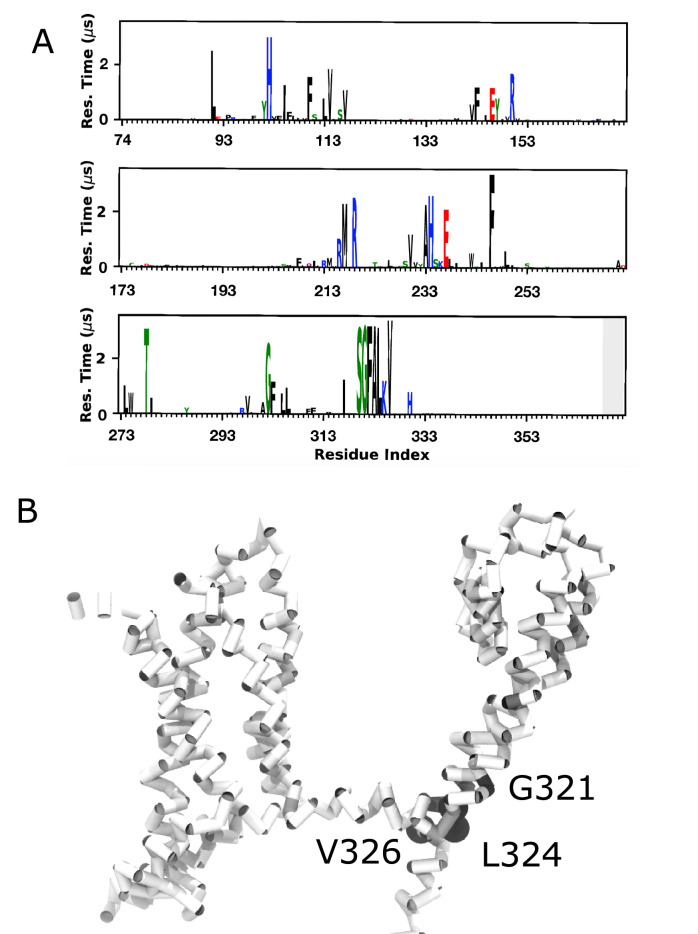
Overview of predicted interaction of uncharged DHA with hK_V_7.4. (**A**) Residence time of DHA projected along the hK_V_7.4 amino acid sequence, calculated from coarse-grained simulations of hK_V_7.4 embedded in a mixture of POPC and neutral DHA. (**B**) Residence times projected as a color scale ranging from white (short residence times) to black (long residence times). Residues with a residence time above 4.4 µs are highlighted as black spheres. Note the absence of a cluster of such residues in the lower half of the VSD. Related to Figure 6.

**Figure 6—figure supplement 3.**
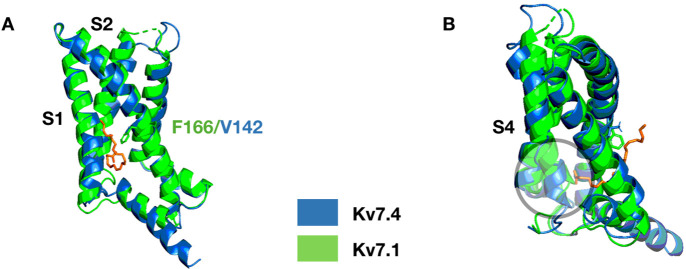
Structural alignment between the VSDs of hK_V_7.1 and hK_V_7.4 highlighting differences in the inner VSD site. (**A**) Structural alignment of the VSDs of hK_V_7.4 (blue, PDB ID – 7BYL) and hK_V_7.1 (green, PDB ID – 6UZZ) highlighting structural differences that may permit DHA binding in hK_V_7.4 but not in hK_V_7.1. F166 (present in hK_V_7.1) can block the entry pathway for DHA (orange sticks), whereas V142 (present in hK_V_7.4) and its smaller side chain permit entry to the unconventional inner site. (**B**) A 90 degree counter-clockwise rotation of the structural alignment in (A) shows a decreased cavity size (highlighted with the black circle) in hK_V_7.1, as the S4 helix of hK_V_7.1 is pushed more into the cavity relative to S4 of K_V_7.4. Related to Figure 6.

### Arginine residues in the lower half of S4 of hK_V_7.4 contribute to the PUFA response

**Figure 7.**
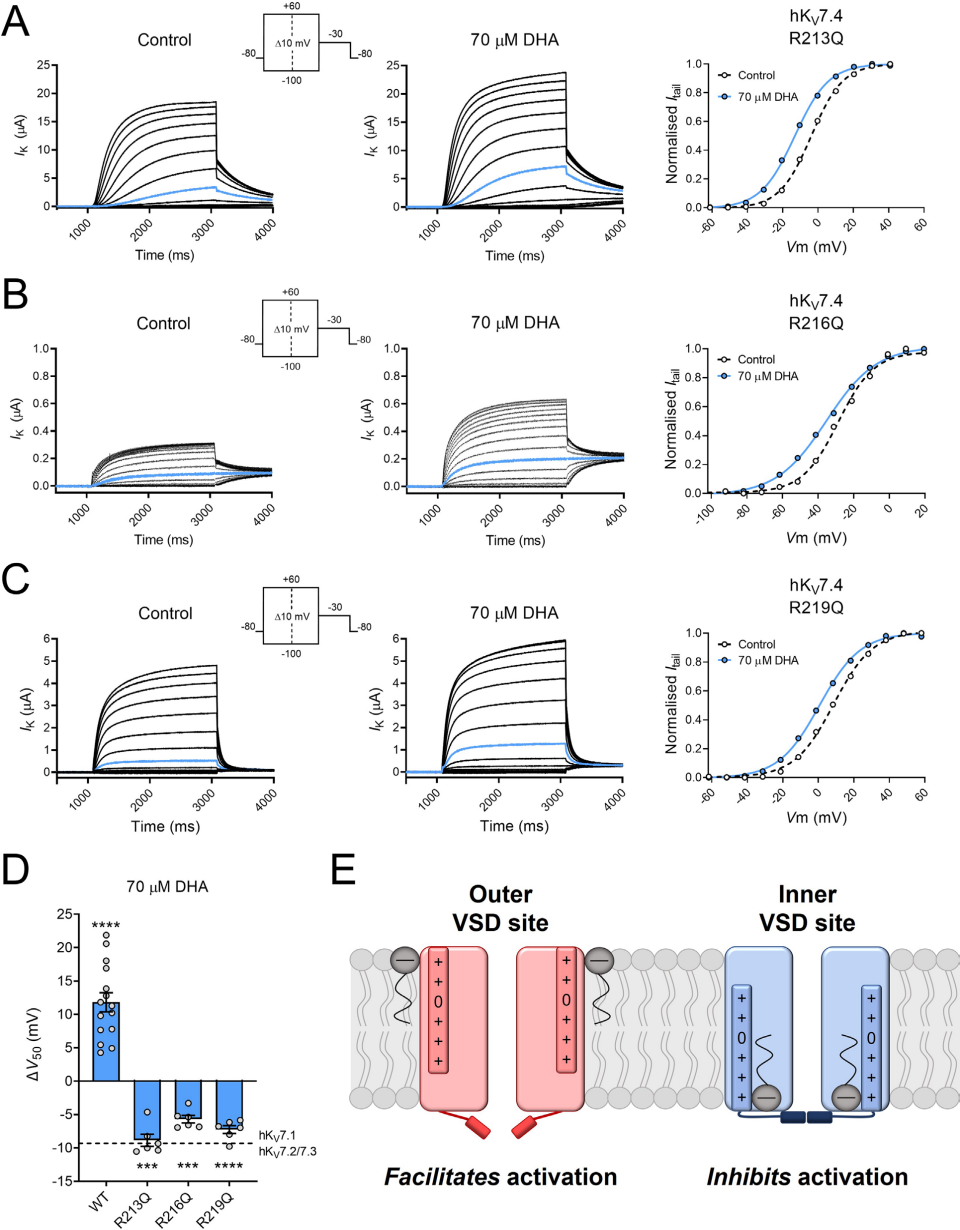
Substitution of innermost S4 arginines with charge-neutral glutamine unmasks PUFA-mediated facilitation of hK_V_7.4 activation. (**A**) Representative current family with corresponding G(V) curve of hK_V_7.4 R213Q in the absence (left) and presence (middle) of 70 µM DHA. Blue traces denote current generated by a test voltage to –10 mV. Curves represent Boltzmann fits. V_50_ for this specific cell: V_50,ctrl_ = –3.8 mV; V_50,DHA_ = –13.2 mV. (**B**) Same as in A but for hK_V_7.4 R216Q. Blue traces denote current generated by a test voltage to –30 mV. V_50_ for this specific cell: V_50,ctrl_ = -28.7 mV; V_50,DHA_ = –35.3 mV. (**C**) Same as in A but for hK_V_7.4 R219Q. Blue traces denote current generated by a test voltage to –10 mV. V_50_ for this specific cell: V_50,ctrl_ = 8.6 mV; V_50,DHA_ = +0.6 mV. (**D**) Summary of the response of hK_V_7.4 gating charge mutants to 70 μM DHA. Data for wild-type hK_V_7.4 has been added for reference. Dashed line indicates the mean ΔV_50_ observed in hK_V_7.1 and hK_V_7.2/7.3 channels exposed to 70 μM DHA (same data as in Figure 5A). Data shown as mean ± SEM. n = 6–15. Statistics denote one sample *t* test against a hypothetical mean of 0 mV. *** denotes p ≤ 0.001, **** denotes p ≤ 0.0001. (**E**) Cartoon illustrating facilitation of channel opening from the conventional outer VSD site (*left*, in red) and inhibition of channel opening from the unconventional inner VSD site (*right*, in blue) mediated by PUFAs (gray). In the outer VSD site, PUFAs interact with the outermost positively charged gating charges of S4, which facilitates channel activation and channel opening. In the inner VSD site, PUFAs interact with the innermost gating charges of S4, stabilizing an intermediate or resting conformation that impairs channel opening. See also Figure 7—figure supplement 1, Figure 7—source data 1. Figure 7—source data 1.Numerical data for Figure 7.

The molecular dynamics simulations were performed using a hK_V_7.4 model based on the cryo-EM structure of hK_V_7.4 (PDB ID - 7BYL), in which S4 is presumably in an intermediate state (Li et al., 2021). S4 is anticipated to move further downward when adopting a resting state and further upward when adopting an activated state. The lack of hK_V_7.4 structure with S4 in a resting state prevents us from using simulations to assess DHA interaction with the inner VSD site in this conformational state. Therefore, to functionally assess the importance of the interactions predicted by the molecular dynamics simulations, we substituted each of the innermost arginines (R213, R216, and R219), one by one, with an uncharged glutamine. This generated mutant hK_V_7.4 channels with V_50_ values shifted toward more positive (R213Q and R219Q) or negative (R216Q) voltages relative to the wild-type channel (Table 2). However, in contrast to the positive shift in V_50_ normally seen in wild-type hK_V_7.4 by DHA, each of these arginine mutants responded to 70 μM DHA with a negative shift in V_50_ (Figure 7A–D, R213Q ΔV_50_ = –8.8 ± 0.9 mV; R216Q ΔV_50_ = –5.7 ± 0.6 mV; R219Q ΔV_50_ = –7.3 ± 0.6 mV). Thus, neutralization of any of these three arginines in the lower half of S4 endowed the hK_V_7.4 channel with a DHA response typical of other hK_V_7 channels (that is, PUFA-mediated facilitation of channel activation), confirming the interaction site predictions from the molecular dynamics simulations. With this typical DHA response, the arginine mutants showed a response more in line with the lipoelectric mechanism, with a negative shift in V_50_ induced by negatively charged PUFAs, no or minimal shift in V_50_ induced by uncharged PUFA analogues, and a positive shift in V_50_ induced by positively charged PUFA analogues (Figure 7—figure supplement 1). This response pattern was clearest for R213Q, which showed the largest negative shift induced by DHA, for which DHA-me had minimal effects on V_50_ (ΔV_50_ = +0.3 ± 0.1 mV, p < 0.05) whereas DHA+ and AA+ shifted V_50_ toward positive voltages (ΔV_50_ = +2.6 ± 0.5 mV, p < 0.005 for DHA+; ∆V_50_ = +3.1 ± 0.4 mV, p < 0.001 for AA+). The response pattern showed a similar trend for R216Q and R219Q, however, with less robust effects induced by the positively charged compounds (For R216Q: ΔV_50_ = +1.6 ± 0.8 mV, p = 0.11 for DHA+; ∆V_50_ = +5.6 ± 2.4 mV, p = 0.06 for AA+; for R219Q: ΔV_50_ = +2.2 ± 1.0 mV, p = 0.08 for DHA+; ∆V_50_ = +4.7 ± 0.7 mV, p < 0.005 for AA+). The positive shifts induced by AA+ on the arginine mutants were larger than for hK_V_7.5 (Figure 3C) and those previously reported for the Shaker K_V_ channel (Börjesson et al., 2010), comparable to the effect on WT hK_V_7.4 (Figure 3F), and slightly smaller than those previously reported for hK_V_7.1 (Liin et al., 2015).

**Figure 7—figure supplement 1.**
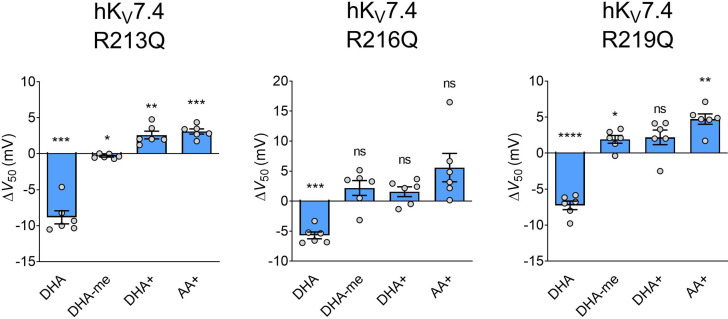
The impact of PUFA head group charge for effects on hK_V_7.4 arginine mutants. Summary of the impact of the head group charge on the ability of PUFAs to shift V_50_ of indicated hK_V_7.4 arginine mutants, assessed by comparing the effects of negatively charged DHA, uncharged DHA-me, and positively charged DHA+ or AA+. Data shown as mean ± SEM. n = 6. Statistics denote one sample *t* test against a hypothetical mean of 0 mV. ns denotes not significant, * denotes p ≤ 0.05, ** denotes p ≤ 0.01, ** denotes p ≤ 0.001, **** denotes p ≤ 0.0001. Related to Figure 7.

## Discussion

This study finds that the activity of hK_V_7.4 and hK_V_7.5 channels is modulated by PUFAs. As such, our study expands upon the understanding of the modulation of the hK_V_7 family of ion channels by this class of lipids. Importantly, we find that both hK_V_7.4 and hK_V_7.5 show surprising responses to PUFAs, compared to previously reported effects on other hK_V_7 subtypes. hK_V_7.4 is the only hK_V_7 subtype for which PUFAs induce channel inhibition, whereas ω–3 PUFAs induce unexpectedly large hK_V_7.5 channel activation. Experiments on co-expressed hK_V_7.4/7.5 channels and hK_V_7.4 channels carrying hK_V_7.5 mimetic disease-associated mutations indicate responses to PUFAs that are intermediate of the hK_V_7.4 and hK_V_7.5 homomers. In contrast, hKCNE4 co-expression did not alter the PUFA response of hK_V_7.4. The diverse responses of hK_V_7 subtypes to PUFAs demonstrate the importance of carrying out functional experiments on each of the channel subtypes to allow for an understanding of subtype variability in channel modulation.

What mechanistic basis may underlie PUFA-induced facilitation of hK_V_7.5 activation? We find that the pattern of how hK_V_7.5 responds to PUFAs conforms to the lipoelectric mechanism that has previously been described for the hK_V_7.1 and hK_V_7.2/7.3 channels, with a hyperpolarizing shift in V_50_ induced by negatively charged PUFAs, a depolarizing shift in V_50_ induced by a positively charged PUFA analogue, and no effect of an uncharged PUFA analogue. Therefore, we find it likely that PUFAs also facilitate activation of hK_V_7.5 through electrostatic interactions with positively charged gating charges in the upper half of the VSD (schematically illustrated in Figure 7E *left*). However, ω–6 PUFAs are less effective than ω–3 PUFAs at activating the hK_V_7.5 channel. This is different from the hK_V_7.1/KCNE1 channel, for which ω–6 or ω–9 PUFAs were identified as the best modulators of channel activity (Bohannon et al., 2019). Although the role of the ω-numbering in determining the extent of PUFA effects on hK_V_7.5 should be interpreted with caution (for instance, because the PUFAs also vary in their number of double bonds, see Table 1), it is clear that hK_V_7.5 and hK_V_7.1/KCNE1 do not follow the same response pattern to different PUFAs. Work on hK_V_7.1/KCNE1 has proposed that the KCNE1 subunit impairs the DHA response of hK_V_7.1 by rearranging one of the external loops in hK_V_7.1, which alters the pH at the outer VSD site of the channel to promote DHA protonation (Larsson et al., 2018; Liin et al., 2015). This led us to test if the greater DHA response of the hK_V_7.5 channel was caused by a lower apparent pKa of DHA at hK_V_7.5 compared to hK_V_7.1–7.3, which might promote DHA deprotonation. However, the similar apparent pKa values of DHA for hK_V_7.1 and hK_V_7.5 discards this hypothesis. Instead, we find a larger magnitude of the DHA effect on hK_V_7.5 at all pH values compared to hK_V_7.1. Interestingly, a residue at the top of S5 of hK_V_7.1, Y278, which was recently identified as an important ‘anchor point’ for binding the ω–6 PUFA LA near the VSD to allow for efficient shifts in V_50_ (Yazdi et al., 2021), is instead a phenylalanine (F282) in hK_V_7.5. A phenylalanine mutation of this ‘anchor point’ in hK_V_7.1 (Y278F) led to a drastic decrease in apparent affinity of LA for hK_V_7.1, possibly by removing hydrogen bonding between the head group of LA and the hydroxyl group of the tyrosine side chain (Yazdi et al., 2021). However, because the Y278F mutation of hK_V_7.1 also reduced the apparent affinity of DHA for hK_V_7.1 (Yazdi et al., 2021), and DHA was the most potent of the PUFAs we tested on hK_V_7.5 in this study, sequence variability at this specific position is not likely to underlie the relatively larger effect of DHA on hK_V_7.5 compared to hK_V_7.1. Thus, we conclude the nature of PUFA binding to hK_V_7 channels is more complex and there may be other stabilizing residues in the PUFA interaction site of the hK_V_7.5 channel.

We find that the activity of the hK_V_7.4 channel is inhibited by PUFAs, and that the direction of the effect is not reversed by reversing the charge of the PUFA head group. Therefore, the PUFA effect on hK_V_7.4 does not conform to the lipoelectric mechanism proposed for other K_V_7 channels. Additionally, we did not find any relationship between PUFA tail properties and the response of hK_V_7.4 to PUFAs, other than that all PUFAs with significant effects shifted V_50_ toward more positive voltages. A cryo-EM structure for hK_V_7.4 was recently reported (PDB ID – 7BYL; Li et al., 2021), which reveals structural differences when comparing to the hK_V_7.1 structure (PDB ID – 6UZZ; Sun and MacKinnon, 2020). For instance, the entire VSD of hK_V_7.4 is rotated 15° clockwise relative to the pore domain of the channel. In molecular dynamics simulations, we also identified an unconventional predicted DHA site in hK_V_7.4. Although the lack of hK_V_7.4 structures in different conformational states limits the ability to simulate the state dependence of DHA interaction with different sites, our experiments validated the functional relevance of the unconventional predicted site. Taken together, our simulation and experimental data suggest that hK_V_7.4 is unique among hK_V_7 channels in harboring a functional PUFA site in the inner half of the VSD. The DHA-induced shift in V_50_ toward positive voltages in wild-type hK_V_7.4 is compatible with DHA stabilizing a resting or intermediate state of S4 through interaction with arginines in the lower half of S4 (schematically illustrated in Figure 7E *right*). Disruption of DHA interaction with this inner VSD site, through substitution of the innermost S4 arginines, endowed hK_V_7.4 with the typical PUFA response of a shift in V_50_ toward negative voltages. We therefore suggest that hK_V_7.4 also harbors a conventional PUFA site at the outer half of the S4, the functional effect of which is unmasked upon disruption of the functionally dominant inner VSD site. In agreement with this, weak DHA interaction with the conventional outer VSD site is indeed observed in our simulations (corresponding to cluster #9, see Figure 6—figure supplement 1B). Furthermore, our experiments using PUFA analogues indicate that it is possible to charge-tune the PUFA response of the hK_V_7.4 arginine mutants from a negative to positive shift in V_50_ by altering the charge of the PUFA head group, a feature which is in line with observations on other K_V_ channels electrostatically modulated by PUFAs (Börjesson et al., 2010; Liin et al., 2015). A structural alignment between the VSDs of hK_V_7.1 (PDB ID – 6UZZ) and hK_V_7.4 (PDB ID – 7BYL) revealed two possible molecular level reasons for the differential binding of DHA to these two channels: in hK_V_7.4 the binding site we uncovered indeed appears both larger, with the S4 helix pushed inwards in hK_V_7.1 relative to hK_V_7.4, and more accessible, as the cleft between S1 and S2 via which DHA appears to enter the cavity features a bulky, obstructing phenylalanine residue (F166) in hK_V_7.1 compared to a smaller valine residue (V142) in hK_V_7.4 (Figure 6—figure supplement 3).

What potential physiological implications may our findings have? Increased consumption of foods rich in PUFAs has long been associated with improved cardiovascular health (Bang et al., 1980; Kagawa et al., 1982; Saravanan et al., 2010) and multiple mechanisms have been proposed, including those mediated by the immune system, the endothelium and vascular smooth muscle tissue (Massaro et al., 2008). Several ion channels in both endothelial cells and VSMCs have been studied as the molecular correlates of PUFA-mediated vasodilatory mechanisms (Bercea et al., 2021). Of note, PUFA-induced inhibition of L-type Ca_V_ channels (Engler et al., 2000) and PUFA-induced activation of large conductance Ca^2+^-activated K^+^ (BK) channels contribute to the vasodilation induced by PUFAs (Hoshi et al., 2013; Limbu et al., 2018). Intriguingly, a recent study by Limbu et al., 2018 observed an endothelium-independent residual relaxation of rodent arteries induced by ω–3 PUFAs despite pre-treatment with several K^+^ channel inhibitors, suggesting there may be another mechanism underlying PUFA-mediated vasorelaxation that does not involve BK channels. Our finding that PUFAs facilitate activation of hK_V_7.5 raises the possibility that K_V_7.5 subunits contribute to the residual vasorelaxation observed by Limbu and colleagues.

Besides cardiovascular effects, an increased intake of ω–3 PUFAs has also indicated a potential protective role against hearing loss (Curhan et al., 2014; Dullemeijer et al., 2010; Gopinath et al., 2010). While these studies propose the protective mechanism of PUFAs on hearing stem from vascular effects that improve cochlear perfusion, no direct mechanism of PUFAs on the hearing organ was investigated. Based on our findings, it would be unlikely that the decreased risk of hearing loss is due to direct actions of PUFAs on K_V_7.4 channels expressed in OHCs, given that we find the channel activity to be inhibited by PUFAs. However, PUFA-induced facilitation of the activity of other K_V_7 channels (e.g. K_V_7.1 and K_V_7.5) in VSMCs could possibly contribute to improved cochlear perfusion. Of note, the two DFNA2-associated missense mutations in hK_V_7.4 we examined, F182L and S273A, showed intrinsic biophysical behavior comparable to that of wild-type hK_V_7.4 (see Table 2). This is in overall agreement with previous studies of these mutants performed in other expression systems showing voltage dependence of channel opening approximate to that of the wild-type channel (Jung et al., 2019; Kim et al., 2011). Jung and colleagues found that the S273A mutant reduces the average whole-cell current densities to less than 50% of the wild-type (Jung et al., 2019), which indicates that S273A may be a risk factor for development of hearing loss through its limited ability to generate K^+^ currents. However, whether F182L acts as a risk factor in DFNA2 hearing loss or should rather be considered a benign missense mutation (Kim et al., 2011) remains to be determined. We find that both the F182L and S273A mutants display an impaired response to DHA, which, if anything, is expected to preserve channel function in the presence of PUFAs.

To conclude, we find that hK_V_7.4 and hK_V_7.5 respond in opposing manners to PUFAs. The hK_V_7.5 channel’s response is largely in line with the responses that have previously been observed in other hK_V_7 channels, whereas the hK_V_7.4 channel response is not. Altogether, our study expands our understanding of how the activity of different members of the hK_V_7 family are modulated by PUFAs and demonstrates different responses explained by different PUFA sites in different hK_V_7 subtypes. Further studies are needed to evaluate putative physiological importance of the effects described in this study in more complex experimental systems.

## Materials and methods

**Key resources table:** 

Reagent type (species) or resource	Designation	Source or reference	Identifiers	Additional information
Gene (human)	KCNQ4	GenBank	Acc.No. NM_004700	
Gene (human)	KCNQ5	GenBank	Acc.No. NM_001160133	
Gene (human)	KCNE4	GenBank	Acc.No. NM_080671	
Chemical compound, drug	Tetradecatrienoic acid (TTA)	Larodan	Cat#: 10–1403	
Chemical compound, drug	Hexadecatrienoic acid (HTA)	Larodan	Cat#: 10–1603	
Chemical compound, drug	Linoleic acid (LA)	Sigma-Aldrich	Cat#: L1012	
Chemical compound, drug	Arachidonic acid (AA)	Sigma-Aldrich	Cat#: A3611	
Chemical compound, drug	Eicosapentaenoic acid (EPA)	Sigma-Aldrich	Cat#: E2011	
Chemical compound, drug	Docosahexaenoic acid (DHA)	Sigma-Aldrich	Cat#: D2534	
Chemical compound, drug	Docosahexaenoic acid methyl ester (DHA-me)	Sigma-Aldrich	Cat#: D2659	
Chemical compound, drug	Indomethacin	Sigma-Aldrich	Cat#: I7378	

### Test compounds

All chemicals were purchased from Sigma-Aldrich, Stockholm, Sweden, unless stated otherwise. The PUFAs used in this study include: tetradecatrienoic acid (TTA, 14:3Δ5,8,11, Larodan, Stockholm, Sweden), hexadecatrienoic acid (HTA, 16:3Δ7,10,13, Larodan, Stockholm, Sweden), linoleic acid (LA, 22:6Δ4,7,10,13,16,19), arachidonic acid (AA, 20:4Δ5,8,11,14), eicosapentaenoic acid (EPA, 20:5Δ5,8,11,14,17), and docosahexaenoic acid (DHA, 22:6Δ4,7,10,13,16,19). PUFA analogues used in this study include: docosahexaenoic acid methyl ester (DHA-me), arachidonoyl amine (AA+), and docosahexaenoyl amine (DHA+). AA+ and DHA+ were synthesized *in house* as previously described (Börjesson et al., 2010). Table 1 summarizes the molecular properties of PUFAs and PUFA analogues used. PUFAs and PUFA analogues were solved in 99.9% EtOH and diluted to their final concentrations in extracellular recording solution. To prevent potential degradation of arachidonic acid, recording solution was supplemented with 5 mM of the cyclooxygenase inhibitor indomethacin. Previous experiments with radiolabeled fatty acids have found that up to 30% of the nominal concentration applied will be bound to the Perspex recording chamber we use (Börjesson et al., 2008). As a result, the effective concentration of freely available fatty acids is 70% of the applied concentration. To allow for comparison with previous studies, we report the effective concentrations throughout the paper.

### Molecular biology

Human KCNQ4 (GenBank accession no. NM_004700), human KCNQ5 (GenBank accession no. NM_001160133) and human KCNE4 (GenBank accession no. NM_080671) were used in this study. cRNA for injection was prepared from DNA using a T7 mMessage mMachine transcription kit (Invitrogen, Stockholm, Sweden), and cRNA concentrations were determined by means of spectrophotometry (NanoDrop 2000c, Thermo Scientific, Stockholm, Sweden). Mutations were introduced through site-directed mutagenesis (QuikChange II XL, with 10 XL Gold cells; Agilent Technologies, Kista, Sweden) and confirmed by sequencing at the Linköping University Core Facility.

### Two-electrode voltage clamp experiments on *Xenopus* oocytes

Individual oocytes from *Xenopus laevis* frogs were acquired either through surgical removal followed by enzymatic digestion at Linköping University, or purchased from Ecocyte Bioscience (Dortmund, Germany). The use of animals, including the performed surgery, was reviewed and approved by the regional board of ethics in Linköping, Sweden (Case no. 1941). Oocytes at developmental stages V-VI were selected for experiments and injected with 50 nL of cRNA. Each oocyte received either 2.5 ng of hK_V_7.4 RNA or 5 ng of hK_V_7.5 RNA. Co-injected oocytes received a 1:1 mix of hK_V_7.4 cRNA (2.5 ng) and hK_V_7.5 cRNA (2.5 ng) for the expression of hK_V_7.4/7.5, or a mix of hK_V_7.4 cRNA (2.5 ng) and hKCNE4 cRNA (1.25 ng) for the expression of hK_V_7.4/KCNE4. Injected oocytes were incubated at 8 °C or 16 °C for 2–4 days prior to electrophysiological experiments.

Two-electrode voltage clamp recordings were performed with a Dagan CA-1B amplifier system (Dagan, MN, USA). Whole-cell K^+^ currents were sampled using Clampex (Molecular devices, San Jose, CA, USA) at 5 kHz and filtered at 500 Hz. For most experiments, the holding potential was set to –80 mV. If experimental conditions allowed for channel opening at –80 mV, the holding potential was set to –100 mV. Current/voltage relationships were recorded using voltage-step protocols prior to and after the application of test compounds. Activation pulses were generated in incremental depolarizing steps of 10 mV, from –100 mV to +60 mV for hK_V_7.4 and hK_V_7.4/KCNE4, from –90 mV to 0 mV for hK_V_7.5, and from –120 mV to +60 mV for hK_V_7.4/hK_V_7.5. The duration of the activation pulse was 2 s for hK_V_7.4, hK_V_7.4/KCNE4 and hK_V_7.4/7.5, and 3 s for hK_V_7.5. The tail voltage was set to –30 mV for all protocols and lasted for 1 s. All experiments were carried out at room temperature (approx. 20 °C). The extracellular recording solution consisted of (in mM): 88 NaCl, 1 KCl, 0.4 CaCl_2_, 0.8 MgCl_2_, and 15 HEPES. pH was adjusted to 7.4 by addition of NaOH. When experiments were conducted at a lower or higher pH, the pH was adjusted the same day as experiments by the addition of HCl or NaOH. Note that the pH of the extracellular recording solution was identical in the control recording and DHA recording of each oocyte. Recording solution containing test compounds was applied extracellularly to the recording chamber during an application protocol (comprised of repeated depolarizing steps every 10 s to 0 mV for hK_V_7.4, hK_V_7.4/KCNE4 or hK_V_7.4/hK_V_7.5 co-injected cells, or to –30 mV for hK_V_7.5) until steady-state effects were observed. Solutions containing PUFAs or PUFA analogues were applied directly and manually to the recording chamber via a syringe. A minimum volume of 2 mL was applied to guarantee the replacement of the preceding solution in the recording chamber. The recording chamber was thoroughly cleaned between cells with 99.5% ethanol.

### Electrophysiological analysis

GraphPad Prism 8 software (GraphPad Software Inc, Ca, USA) was used for data analysis. The voltage-dependence of hK_V_7 channels was approximated by plotting the immediate tail currents (recorded upon stepping to the tail voltage) against the preceding test voltages. Data were fitted with a Boltzmann function, generating a G(V) (conductance *versus* voltage) curve:

$$G_{\left(V\right)}=G_{min}+\frac{\left(G_{max}-G_{min}\right)}{\left[1+exp\left(\frac{V_{50-x}}{s}\right)\right]}$$

where G_min_ is the minimum conductance, G_max_ is the maximum conductance, V_50_ is the midpoint of the curve (i.e, the voltage determined by the fit required to reach half of G_max_) and s is the slope of the curve. The difference between V_50_ under control settings and under test settings for each oocyte (the ΔV_50_) was used to quantify shifts in the voltage-dependence of channel opening evoked by test compounds. The relative difference between G_max_ under control settings and under test settings for each oocyte (the ΔG_max_) was used to quantify changes in the maximum conductance evoked by test compounds. Note that representative G(V) curves have been normalized in figures to allow for better visualization of V_50_ shifts.

To determine the concentration dependence or the pH dependence of ΔV_50_, the following concentration-response function was used:

$$\Delta V_{50}=\frac{\Delta V_{50,max}}{\left[1+{\left(\frac{EC_{50}}{C}\right)}^{N}\right]}$$

where ΔV_50,max_ is the maximum shift in V_50_, C is the concentration of the test compound, EC_50_ is the concentration of a given test compound or the concentration of H^+^ required to reach 50% of the maximum effect, and N is the Hill coefficient (set to 1 or –1). For studying pH dependence, the values of C were determined with asymptotic 95% confidence intervals in GraphPad Prism 8 and subsequently log-transformed to acquire the apparent pKa values.

### Coarse-grained molecular dynamics simulations

The full-length cryo-EM structure of hK_V_7.4 channel in an intermediate activation state (PDB ID - 7BYL) (Li et al., 2021) was prepared by building in missing residues using the MODLOOP webserver (Fiser et al., 2008). The channel was embedded in a heterogenous bilayer consisting of 480 1-palmitoyl-2-oleoyl-sn-glycero-3- phosphocholine (POPC) molecules and 120 docosahexaenoic acid (DHA) molecules (placed at random and distributed equally in both the leaflets) using the CHARMM-GUI MARTINI (Bilayer system) maker (Qi et al., 2015). In one system, DHA with a negatively charged head group was used while in the other, DHA with a neutral head group was considered. The systems were then solvated by adding a~45 Å layer of water to each side of the membrane. Lastly, systems were ionized to reach a 150 mM KCl concentration. Martini 2.2 force field combined with ElNeDyn (Elastic network in dynamics) was used. The systems were minimized and equilibrated following the default CHARMM-GUI protocol. During equilibration, pressure was maintained at 1 bar through Berendsen pressure coupling; temperature was maintained at 300 K through velocity rescaling thermostat (Bussi et al., 2007) with the protein, membrane and solvent coupled. During production simulation, pressure was maintained at 1 bar through Parinello-Rahman pressure coupling (Parrinello and Rahman, 1981). A time step of 20 fs was used. Finally, 19 µs production simulations were carried out for the system with neutral DHA, while eight 5 µs simulations were run for the system with negatively charged DHA. Simulations were performed using GROMACS version 2020.4 (Abraham et al., 2015). The analysis of the coarse-grained simulations was carried out using PyLipid (Song et al., 2022). Frames extracted every 20 ns were used for the analysis. A dual cut-off scheme (lower limit: –0.5 nm, upper limit: –0.7 nm) was used to determine the interactions between DHA and the protein. Interactions were averaged across all subunits of the protein. Binding regions were determined by identifying clusters of at least four residues that interact with the same DHA molecule at the same time. These residues were found by community analysis of residue interaction networks. The most representative bound pose for each binding site was extracted by a scoring of the bound poses based on a density based scoring function, where the probability density is calculated from the simulation trajectories.

### Illustrations

For the structural images shown in Figure 4, the cryo-EM resolved structure of the hK_V_7.4 channel (PDB ID - 7BYL [Li et al., 2021]) was visualized using The Protein Imager (Tomasello et al., 2020). Visualizations of the MD simulation systems were done using Visual Molecular Dynamics (VMD) (Humphrey et al., 1996).

### Statistical analysis

Average values are expressed as mean ± SEM. When comparing two groups, a Student’s *t* test was performed. One sample *t* test was used to compare an effect to a hypothetical effect of 0 (for ΔV_50_) and 1 for (ΔG_max_). When comparing multiple groups, a one-way ANOVA was performed, followed by Dunnett’s multiple comparison test when comparing to a single reference group. A p-value <0.05 was considered statistically significant. All statistical analyses were carried out in GraphPad Prism 8.

## Data Availability

Numerical data is provided in figures and/or corresponding figure legends, Table 2, and the source data files. Source data for molecular dynamics simulations is accessible at https://osf.io/6fuqs/. The following dataset was generated: DelemotteL ChoudhuryK 2022Kv7 PUFAsOpen Science Framework6fuqs

## Additional files

Transparent reporting form
